# TRIB3 is a biomarker of poor prognosis in laryngeal squamous cell carcinoma and may affect tumor development through PI3K / AKT / mTOR pathway

**DOI:** 10.1016/j.clinsp.2025.100576

**Published:** 2025-01-26

**Authors:** Runsheng Yuan, Zhongqiang Cheng, Xiaodong Zhan

**Affiliations:** Department of Otolaryngology and Head and Neck Surgery, The First Affiliated Hospital of Bengbu Medical College, Anhui Province, China

**Keywords:** TRIB3, Laryngeal Squamous Cell Carcinoma, Prognosis, Biomarkers, Immunotherapy

## Abstract

**Objective:**

TRIB3 has been confirmed to participate in and regulate biological metabolic activities in head and neck tumors such as nasopharyngeal carcinoma and oropharyngeal carcinoma, so the purpose of this study was to explore whether there is a correlation between TRIB3 and Laryngeal Squamous Cell Carcinoma (LSCC) and to preliminarily explore the biological characteristics of TRIB3 in LSCC.

**Methods:**

TRIB3 expression in the LSCC was analyzed based on The Cancer Genome Atlas (TCGA) database. CCK-8 assay, Colony Formation Assay, wound healing assay, and Transwell assay were performed to investigate the roles of TRIB3 in the proliferation, invasion and metastasis of LSCC. The Protein-Protein Interaction (PPI) network of TRIB3 was analyzed based on the STRING database. Western blotting and qRT-PCR were employed to detect the protein and mRNA expression of TRIB3.

**Results:**

TRIB3 over-expressed in the LSCC, which was related to the poor prognosis of LSCC. Patients with methylation related to high TRIB3 expression had a poorer prognosis. Knock-down of TRIB3 expression suppresses the growth, invasion and migration of LSCCs via PI3K / AKT / mTOR. TIME analysis, surface checkpoint analysis, and prediction model indicated that TRIB3 related risk model displayed a poor prognosis.

**Conclusion:**

This study investigates the role of TRIB3 in the biological characteristics of LSCC, and preliminarily concludes that TRIB3 may be a potentially promising prognostic biomarker for LSCC. TRIB3 may facilitate the growing, invasive and migrating LSCC, leading to a poor prognosis.

## Highlight

• TRIB3 over-expression was related to the poor prognosis of LSCC.
• Patients with methylation related to high TRIB3 expression had a poorer prognosis.
• Knock-down of TRIB3 expression suppresses the growth, invasion and migration of LSCCs via PI3K- AKT- mTOR.
• TRIB3 may be viewed as a possible cancer biomarker.

## Introduction

Squamous Cell Carcinoma of the Larynx (LSCC) is one of the more common types of malignancies of the head and neck.^1^ The screening, diagnosis, and treatment of LSCC have been continuously precise in recent years.^2^ However, existing studies report that the diagnosis of early-stage LSCC and the five-year Overall Survival time (OS) of patients with LSCC have not improved significantly.^3^ Therefore, it is important to screen new biomarkers of LSCC and explore the potential pathogenesis of LSCC, which are helpful for the improvement of clinical efficacy and prognosis.

Tribbles homolog 3 (TRIB3) is a signal transduction mediator and scaffold protein^4^ and plays a “stress adjusting switch” role in the pathogenesis of lung cancer, breast cancer, colorectal cancer, and other cancers.^5^ It can control the growth and metastasizing of the cells of cancer.^6^ In malignant tumors of the head and neck (such as oropharyngeal cancer and nasopharyngeal carcinoma), TRIB3 can function to regulate cell functions via PI3K / AKT / mTOR signaling pathway. It can regulate glucose metabolism in cells to accelerate the occurrence and development of cancers.^7^ To date, no studies have been conducted to investigate whether TRIB3 can further affects the LSCC onset development and prognosis by regulating the PI3K / AKT / mTOR pathway.

In recent years, the use of immunotherapies in the treatment of malignancies has become increasingly widespread such as gastric cancer, prostate cancer and others, achieving favorable efficacy.^8^ Immune therapy modulates the immunity of the host and the tumor microenvironment to kill cancer cells and achieve a therapeutic effect.^9^ The Tumor Microenvironment (TME) is the environment linked to tumor development and progression and is essential for cancer cell development, growth, invasion as well as migration.^10^ However, the immunological mechanism and TME in the pathogenesis of cancers should be further elucidated. Hence, It is essential to recognize very sensitive bio-markers or target genes for LSCC based on molecular biology and genetics.

The purpose of the current research was to examine TRIB3 gene level expression in the LSCC and its relationship with the biometric characteristics of LSCC, The biological features of LSCC include cell proliferation, invasion, migration, etc., which may give a theory base for the research of pathogenesis and immunotherapy of LSCC.

## Methods

### Clinical sampling, cell cultures and transfections

#### Clinical sampling, cells, and reagents

Procedures in this research were all conducted in compliance with the Declaration of Helsinki, and this research was approved by the Ethical Review Committee of the First Hospital of Bengbu Medical College (2022KY075). The study conforms to the STROBE statement. Collection of surgical specimens from 4 LSCC patients and all specimens are kept in liquid nitrogen. Each patient was given informed consent prior to the research. Cells including Tu-212, Tu-686, Tu-177, and 16HBE cells were bought from iCell Bioscience Inc, Shanghai. sh-TRIB1-3, reverse transcription kit and total RNA extraction kit were from Shanghai Genepharma Co., Ltd. RPMI-1640 and trypsin were from Grand Island Biological Company (USA) and Materigel were from TianHang Biotech Co., Ltd (Zhejiang, China). Lipofectarn 2000 (Thermo Scientific, USA), the primary antibody against TRIB3 (Affinity, USA), primary antibodies against GAPDH, PI3K, AKT and mTOR and rabbit secondary antibody (Abbkine Co., Ltd, USA)were also used in this research.

#### Cell culture, transfection and grouping

Cell transfection knockdown TRIB3 for subsequent experiments such as cytopathology and pathway protein experiments.16HBE, Tu-212, Tu-686, and Tu-177 cells were thawed at 37 °C. Tu-212 cells were in BL312A-IMDM; Tu-177 and 16HBE cells were grown in RPMI-1640; Tu-686 cells were grown in high glucose DMEM. When the cells have arrived at 80 %‒90 % convergent, they are transmitted. After digestion with 0.25 % trypsin for 3 min, Tu-212, Tu-686, Tu-177and 16HBE cells were inoculated onto 6-well plates. TRIB3 (shRNA1–3) and shRNA NC were used to transfect Tu-212 cells, Tu-686 cells, Tu-177 cells, and 16HBE cells for 6‒8 h in the presence of Lipofectamine 2000. Then, the medium was refreshed, and the transfection efficiency was assessed. Based on experimental results, Tu-212 and Tu-686 cells were used in the experiment below. shRNA NC served as a control; shRNA TRIB3 had the highest transfection efficiency and was then used in the following experiments.

### Acquisition of TRIB3 expression profile

Bioinformatics website was used to analyze the expression level of TRIB3 in LSCC. In the Oncomine database (https://www.oncomine.org/resource/login.html), the “Gene Differential Expression” section was selected to perform the assessment of TRIB3 expression in LSCC.LSCC data were downloaded from TCGA database (https://www.cancer.gov/) (*p-value < 0.05; *t*-test). Paired and unpaired differential analysis of TRIB3 expression in laryngeal cancer tissues and normal tissues was determined using the “limma” R package (*p-value < 0.05; *t*-test) through the Kaplan-Meier Plotter online survival analysis website (https://kmplot.com/analysis/), selected Select the “LSCC” module to analyze TRIB3 expression in LSCC compared with OS (Overall Survival, overall survival Period; *n* = 217) of the correlation. According to the average value of TRIB3 expression, the database patient samples were divided into high and low expression groups (OS: high expression group = 124, low expression group = 93) (^⁎⁎⁎^p-value < 0.001; *t*-test).

### Gene set enrichment analysis (GSEA) pathway enrichment

Gene enrichment assay was used to explore the TRIB3-related cell signaling pathway in LSCC. To explore the pathway of TRIB3 in LSCC. GSEA version 4.1.0, was used to determine the relationship with TRIB3 in LSCC. The uplinked gene set was identified as type h (h = 28/52) and the downlinked gene set was identified as type l (*l* = 4/12) and “h-versus-l” was chosen to be enriched with the TRIB3-associated route.

### Real time fluorescence quantitative PCR

The expression of TRIB3 mRNA in cells was detected by qRT-PCR assay to evaluate the transfection effect. LSCC cells were cleansed with PBS. After treatment with Trizol reagent, cells were subjected to RNA extraction, and then cDNA was synthesized with total RNA. cDNA (2 μL) was subsequently used for the Real-time PCR (25 μL): ×SuperReal Premix (12.5 μL); primers (10 μmoL/L; 0.75 μL for each); RNase-free ddH_2_O (9 μL). GAPDH served as an interior guide, and 2^−△△^*^Ct^* probed approaches used for TRIB3 mRNA expression were employed for the assessment of transfection efficacy (Table 1). This experiment was performed in triplicate.

**Table 1:** Primers used for qRT-PCR.

Gene	Forward primer	Reverse primer
TRIB3	5′-GCTCCCTTCTAAAATAACAA-3′	5′-TTTTGTCAGATACCTTGGACTTG-3′
GAPDH	5′-TCAAGATCATCAGCAATGCC-3′	5′-CGATACCAAAGTTGTCATGGA-3′

### Wound healing assay

Wound wound-healing assay was used to examine the migratory effects of cells. After transfection, LSCC cells were sown to 6-well plates, followed by incubation. Then, they made a cut with a 10 µL needle, and the cell migration was assessed immediately or 24 h later under a light microscope. In brief, 3‒5 fields were randomly selected, and the width of the wound was measured. This experiment was performed in triplicate. This experiment was performed in triplicate.

### Transwell assay

#### Migration assay

A migration assay was used to examine the migration effect of the cells. After transfection, LSCC cells were sown to the top compartment was kept at an intensity of 1 × 10^5^ in the supernatant medium. The down compartment has a medium mixture involving 10 % FBS. Cells are given permission to emigrate from the upper chambers to the lower chambers within 24 h The top compartments were harvested, and cleaned with PBS. After air drying, the cell migration was assessed. This experiment was performed in triplicate.

#### Invasion assay

Invasion assay was used to examine the Invasion effect of the cells. The chambers pre-coated with Materigel were used for the invasion assay. After transfection, LSCC cells were seeded into upper chambers pre-coated with Materigel at an intensity of 1 × 10^5^, and then grown in supernatant medium. Cells are given permission to emigrate within 48 h The top compartments were harvested, and cleaned with PBS. After air drying, the cell migration was assessed. This experiment was performed in triplicate.

### Colony formation assay

Colony Formation assay was used to examine the proliferation effect of the cells. After transient transfer, LSCC cells were sown to 6-well boards to an intensity of 1500 cells / 2 mL/well and maintained in a medium mixture involving 10 % FBS. The medium was refreshed once every 2‒3 days. The colonies were counted in each well under a microscope. This experiment was performed in triplicate.

### Detection of cell proliferation by CCK-8 assay

CCK-8 assay was used to examine the proliferation effect of the cells. The CCK-8 Detection Assay Test Kit was utilized to determine cell prosthesis. After transient transfer, LSCC cells were sown into 96-well panels (2000 well) and then cultivated for 0h , 24h , 48h and 72 h CCK-8 solvent (10 μL) was then added to every hole, followed by incubation by 1 hour. The Optic Densities (OD) of every hole were calculated with a microboard viewers and growth profiles were plotted to reflect the multiplication of LSCC at 0h , 24h , 48h and 72 h This experiment was performed in triplicate.

### Analysis of TRIB3-associated immunomodulators in the TME of LSCC

Using the “immunomodulators” panel of TISID laryngeal cancer TRIB3-related immunomodulators, the authors analyzed the association of 23 immunosuppressive and 44 immune stimulant levels with TRIB3 expression (*p-value < 0.05). Then, a protein interaction network of immunomodulators associated with TRIB3 expression was constructed using the interaction gene/protein retrieval search tool (string) website (https://www.string-db.org/online). Next, the authors performed a functional analysis of TRIB3-related immunomodulators using mettp://p.org/gp/index.html/main/step1). Based on the immunomodulators associated with TRIB3 expression, the authors used the minimum absolute Shrinkage and Selection Operator Regression (LASSO) algorithm to perform penalty parameter adjustment by 10-fold cross-validation with the “glmnet” and “Survival” R packages. Then, a stepwise multivariate Cox proportional hazards regression analysis of the lasso-identified immunomodulators was performed to determine the optimal immunomodulatory capacity.

### Statistical analysis

Data were statistically analyzed using SPSS version 26.0. Data were expressed as mean ± normal variation. Comparisons between groups were made employing one-way ANOVA and comparisons were made using *t*-tests. Survival rates were determined employing the Kaplan-Meier method.

## Results

### TRIB3 is linked to poor prognosis of LSCC

**Fig. 1:**
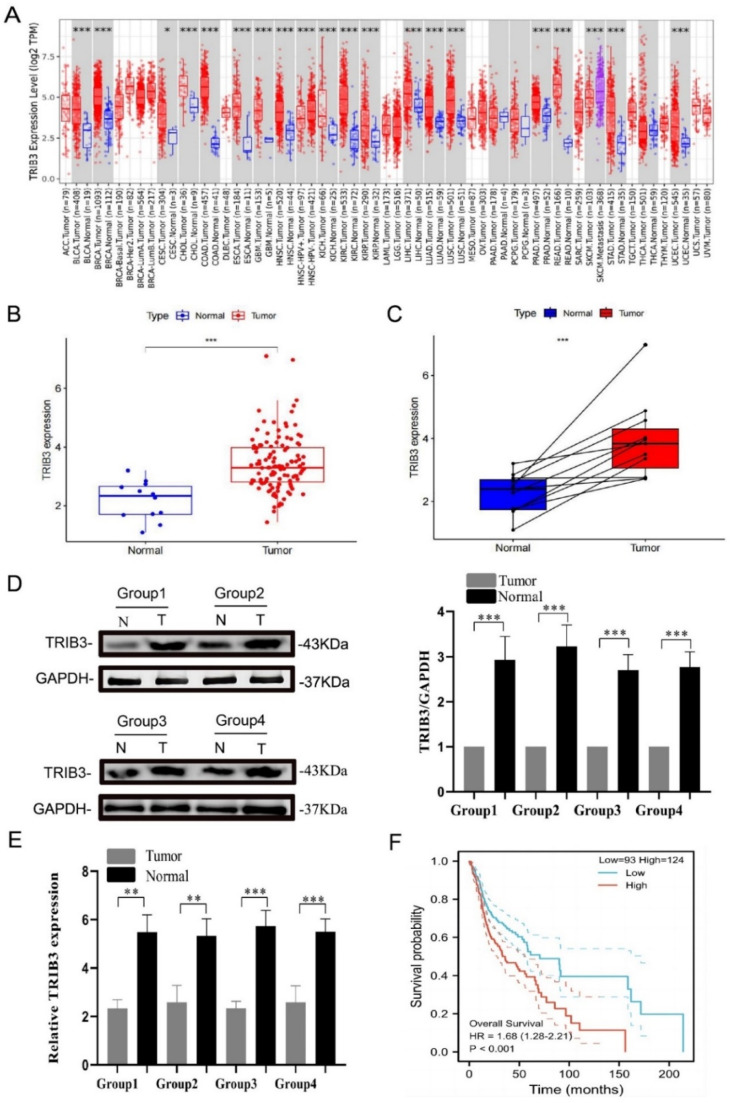
TRIB3 expression in TCGA database and LSCC tissues. (A) In the TCGA database, pan-cancer analysis showed TRIB3 was over-expressed in the LSCC; (B‒C) In the TCGA database, paired difference analysis and unpaired difference analysis showed TRIB3 was over-expressed in the LSCC; (D‒E) Detection of TRIB3 expression in the LSCC tissues and adjacent normal tissues by Western blotting and qRT-PCR. Results showed TRIB3 over-expression in the LSCC tissues, *n* = 4; (F) Kaplan-Meier method was employed to delineate survival curve (OS) based on the TCGA database, and results showed TRIB3 was related to the prognosis of LSCC (OS), n_low_ = 93 and n_high_ = 124 (** p-value < 0.01;*** p-value < 0.001).

Analysis of TCGA database showed TRIB3 over-expression in LSCC (*** p-value < 0.001; Fig. 1A). Unpaired difference analysis showed TRIB3 expansion in LSCC is at a substantially higher level than in adjacent normal tissues, which is in accordance with the findings of the pairwise difference analysis (*** p-value < 0.001; Fig. 1B‒1C). To verify the overexpression of TRIB3 in LSCC, the protein and mRNA of TRIB3 were verified in LSCC tissues and neighboring tissues from 4 patients by Western blotting and qRT-PCR, respectively. Results confirmed that the TRIB3 levels rose significantly in the LSCC tissues than in neighboring tissues (** p-value < 0.01; *** p-value < 0.0 01; Fig. 1D‒E). The Overall Survival Analysis Curves Results showed Samples with high TRIB3 levels had markedly shorter survival times than low TRIB3 expansion (*** p-value < 0.001; Fig. 1F). This indicates that TRIB3 may affect the prognosis of LSCC patients.

#### Methylation sites of TRIB3 gene and its relationship with prognosis of LSCC

**Fig. 2:**
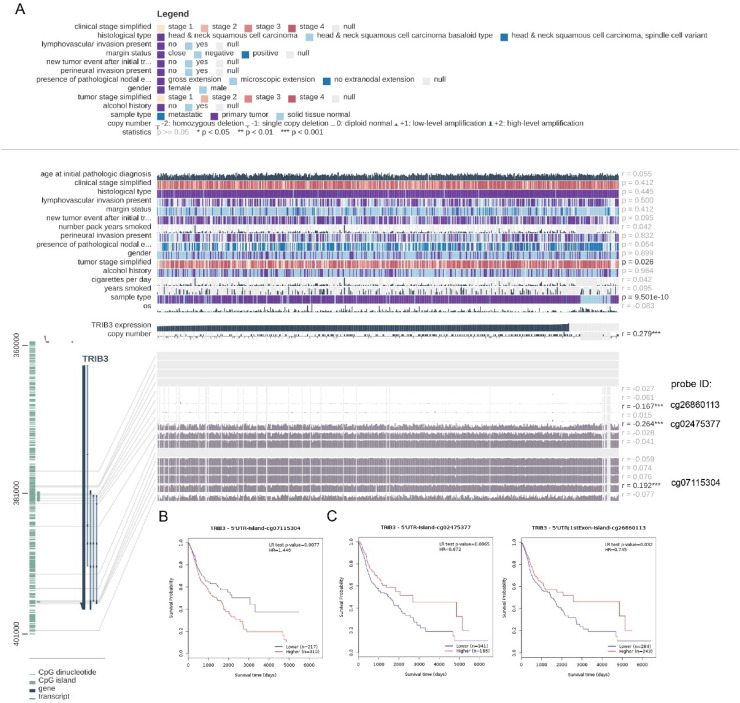
Correlation analysis of methylation sites of TRIB3 gene and prognosis analysis. (A) Methylation sites related to the expression of TRIB3; (B) positive methylation sites of TRIB3: survival rate in high expression group was significantly lower than in low expression group; (C) Negative methylation sites of TRIB3: survival rate in high expression group was markedly higher than in low expression group (r, Correlation; probe ID, Methylation site; * p-value < 0.05; ** p-value < 0.01; *** p-value < 0.001).

Among the methylation sites of TRIB3 gene, cg07115304 was positively related to the TRIB3 expansion (*r* > 0, * p-value < 0.05); cg02475377 with cg26860113 were adversely related to the TRIB3 expansion (*r* < 0, * p-value < 0.05; Fig. 2A). survivalmeth found 3 methylation sites of TRIB3 were related to the survival. cg07115304 highly exploited samples have a poorer prognosis (* p-value < 0.05; Fig. 2B). cg02475377 and cg26860113 lowly exploited samples have a poorer prognosis (* p-value < 0.05; Fig. 2C). These findings were consistent with previous results that high expansion of methylation sites of TRIB3 predicts a poorer prognosis of LSCC.

### Knock-down of TRIB3 expression inhibits biological characteristics of LSCC cells

**Fig. 3:**
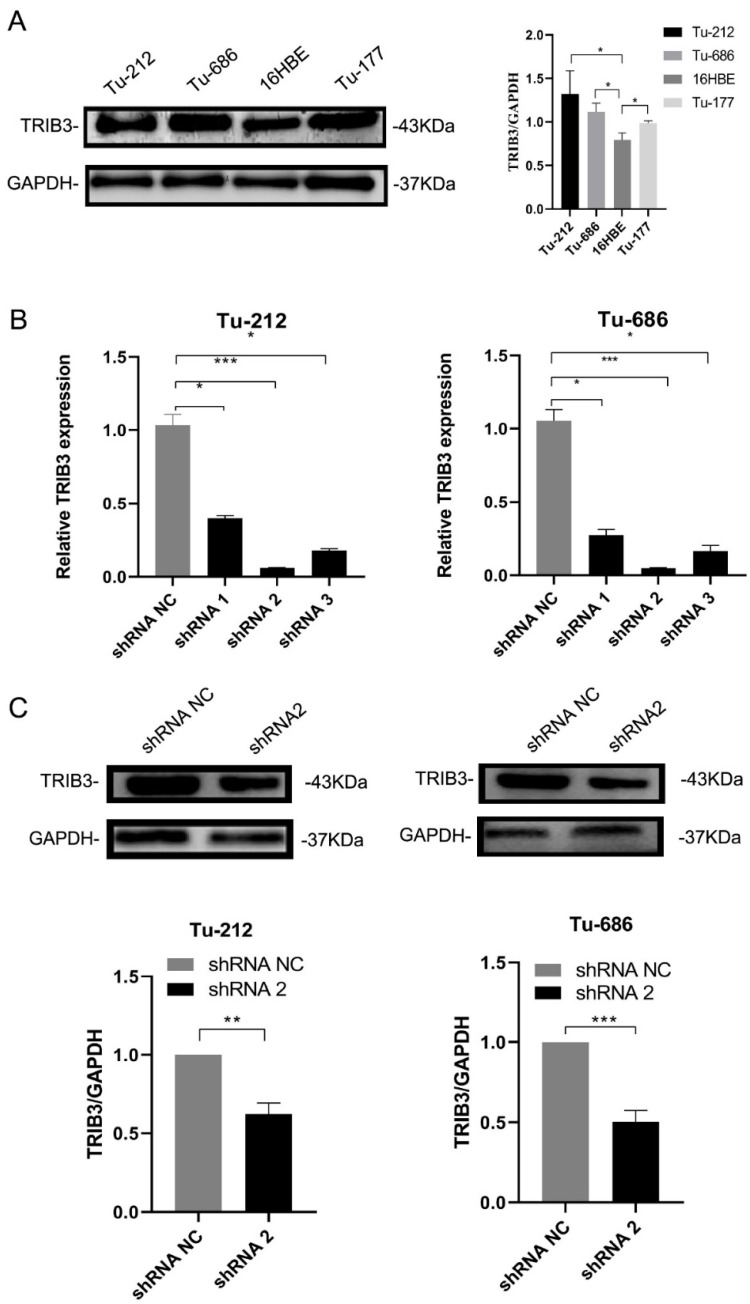
Down-regulation of TRIB3 in different LSCC cell lines. (A) Western blotting was employed to detect TRIB3 expression in different LSCC cell lines. Results showed the TRIB3 expression was the highest in the Tu-686 cells and Tu-212 cells (*p-value < 0.05); (B) Cells were transfected with shRNA targeting TRIB3, and qRT-PCR was employed to detect TRIB3 expression. Results showed the TRIB3 expression was the lowest in LSCC cells after transfection with shRNA2 (** p-value < 0.01; *** p-value < 0.001); (C) Western blotting was employed to detect the protein expression of TRIB3 in LSCC cells after transfection with shRNA2, and results showed the TRIB3 expression in the LSCC cells transfected with shRNA2 was markedly lower than in cells transfected with shRNA NC.

To explore the extent of TRIB3 in 16HBE, Tu-212, Tu-686, and Tu-177, the authors applied a Western blotting assay and the findings displayed The TRIB3 exposure was obviously greater in LSCC cells than in 16HBE cells (* p-value < 0.05; Fig. 3A). Based on this result and cell culture, Tu-212 cells and Tu-686 cells were used in the following experiments. shRNA was used to down-regulate TRIB3 expansion in LSCC cells and qRT-PCR was applied to discover TRIB3 expansion (Table 2). Results showed shRNA2 had the most potent capability to down-regulate TRIB3 expansion (* p-value < 0.05; ** p-value < 0.01; *** p-value < 0.001; Fig. 3B). Western blotting results confirmed that the amplification of TRIB3 in LSCC cells transduced with shRNA2 was significantly lower than that in untransduced (* p-value < 0.05; ** p-value < 0.01; *** p-value < 0.001; Fig. 3C).

**Table 2:** shRNA sequences.

Gene	Sense	Antisense
shRNA1 (hTRIB3–1085)	5′-GAUCUCAAGCUGUGUCGCUUUTT-3′	5′-AAAGCGACACAGCUUGAGAUCTT-3′
shRNA2 (hTRIB3–608)	5′-GACAACUUAGAUACCGAGCGUTT-3′	5′-ACGCUCGGUAUCUAAGUUGUCTT-3′
shRNA3 (hTRIB3–1510)	5′-CCCAACCCGAUCCCAUCUCUGTT-3′	5′-CAGAGAUGGGAUCGGGUUGGGTT-3′

**Fig. 4:**
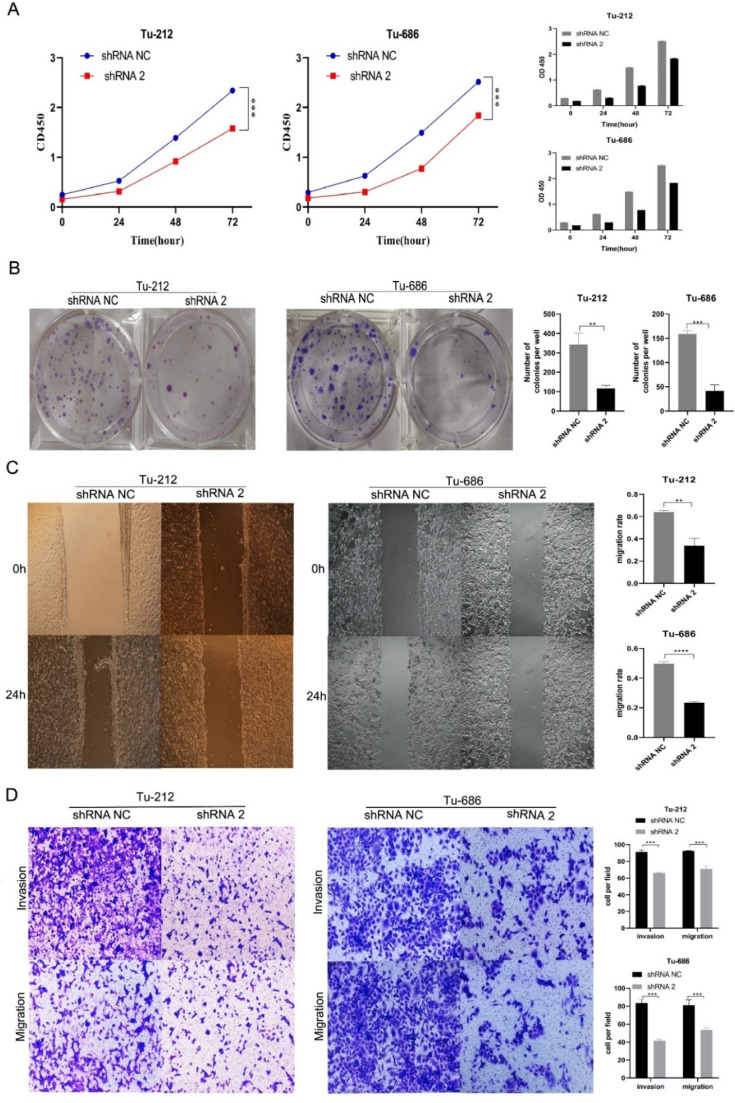
Effect of TRIB3 on the in vitro proliferation and migration of LSCC cells. (A) CCK-8 assay was performed to detect the proliferation of two LSCC cell lines. Results showed down-regulation of TRIB3 inhibited the growth of Tu-212 and Tu-686; (B) Colony formation assay showed the number of colonies formed in LSCC reduced significantly after down-regulation of TRIB3 expression; (C) Wound healing assay showed the migration capability of LSCC reduced significantly after down-regulation of TRIB3; (D) Transwell assay was employed to assess the invasion and migration of LSCC. Results indicated the invasion and migration of LSCC reduced dramatically after down-regulation of TRIB3 (** p-value < 0.01; *** p-value < 0.001).

Cell multiplication test (CCK-8 test) and colony formation assay test findings displayed the multiplication of LSCC cells with down-regulation of TRIB3 expression was significantly inhibited as compared to shRNA NC treated LSCC (*** p-value < 0.001; **** p-value < 0.0001; Fig. 4A‒4B). Wound healing showed the migration of LSCC cells transfected with shRNA2 was significantly inhibited as compared to shRNA NC treated. The TRIB3 on migrating and invading LSCC cells was also inspected by transmissionwell assay. Findings displayed LSCC cells transfected with shRNA had markedly reduced migrating and invading as compared to the shRNA NC treated cells (*** p-value < 0.001; Fig. 4C‒D). These findings indicate that downregulated TRIB3 expansion can restrain the in vitro growth, migrating and invasion of LSCC.

### TRIB3 modulates cellular signaling pathways and affects LSCC metabolism

**Fig. 5:**
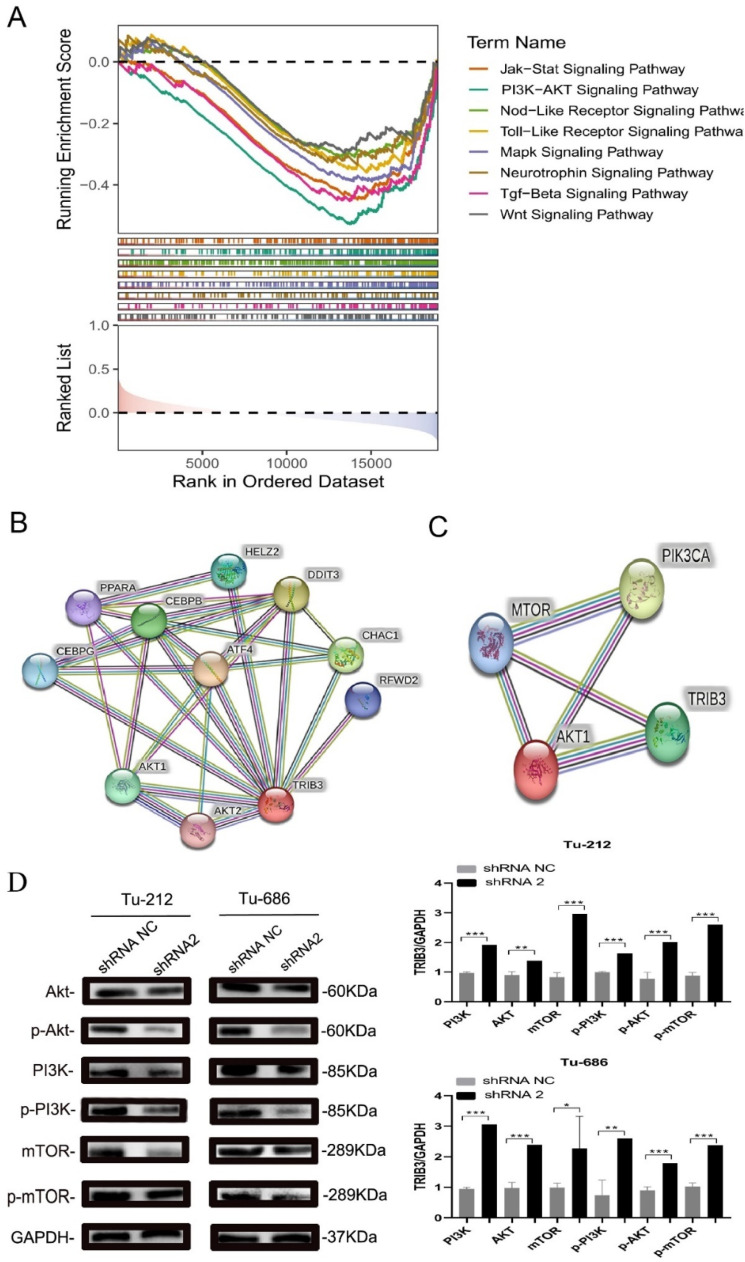
TRIB3 regulates PI3K /AKT /mTOR pathway to affect the invasion and migration of LSCC cells. (A) GSEA pathway enrichment analysis showed TRIB3 enriched pathways included PI3K / AKT / mTOR pathway; (B) PPI analysis showed the downstream factors of TRIB3 included the members of AKT family; (C) PPI analysis showed PI3K / AKT / mTOR pathway mediated the relationship between TRIB3 and AKT; (D) Western blotting showed down-regulation of TRIB3 expression inhibited the expression of PI3K, AKT and mTOR (* p-value < 0.05, ** p-value < 0.01, *** p-value < 0.001).

Studies have reported that TRIB3 can regulate cell metabolism via PI3K/AKT/mTOR circuit for liver cancer, oral and nasopharyngeal carcinoma.^11^ Thus, the authors speculated that TRIB3 could regulate PI3K / AKT / mTOR circuit to Impact the growing, migrating, and invading of LSCC. GSEA enrichment was employed to analyze the enrichment of TRIB3 in LSCC. Results showed the enriched pathways in LSCC mainly included PI3K / AKT / mTOR pathway, Wnt pathway, Jak/stat pathway, and others (Fig. 5A and Table 3). Then, PPI network analysis was performed for TRIB3 based on the STRING database. Results showed AKT protein family was closely related to TRIB3, and TRIB3 could target AKT1 to regulate PI3K /AKT / mTOR pathway (Fig. 5B‒5C). Moreover, the protein production of total and phosphorylated PI3K, AKT, and mTOR was detected in LSCC. Results showed, that in the LSCC cells with down-regulation of TRIB3 expression, the protein expression of PI3K, AKT, mTOR, p-PI3K, p-AKT, and p-mTOR was markedly lower than in shRNA NC (** p-value < 0.01; *** p-value < 0.001; **** p-value < 0.0001; Fig. 5D). This suggested that downregulation of TRIB3 excitation may regulate the PI3K / AKT / mTOR pathway to inhibit the malware phenomenon of LSCC.

**Table 3:** Visualization of gene enrichment.

GeneSet	NES	NOM p-val	FDR q-val
WNT_SIGNALING_PATHWAY	1.91	0.0000	0.0054
NOD_LIKE_RECEPTOR_SIGNALING_PATHWAY	1.89	0.0000	0.0057
NEUROTROPHIN_SIGNALING_PATHWAY	1.90	0.0000	0.0058
MAPK_SIGNALING_PATHWAY	1.91	0.0000	0.0061
JAK_STAT_SIGNALING_PATHWAY	1.85	0.0000	0.0069
HEDGEHOG_SIGNALING_PATHWAY	1.87	0.0000	0.0069
TOLL_LIKE_RECEPTOR_SIGNALING_PATHWAY	1.84	0.0000	0.0069
TGF_BETA_SIGNALING_PATHWAY	1.84	0.0000	0.0070
PI3K_AKT_SIGNALING_PATHWAY	1.82	0.0000	0.0079

### Functional analysis of TRIB3 in LSCC

**Fig. 6:**
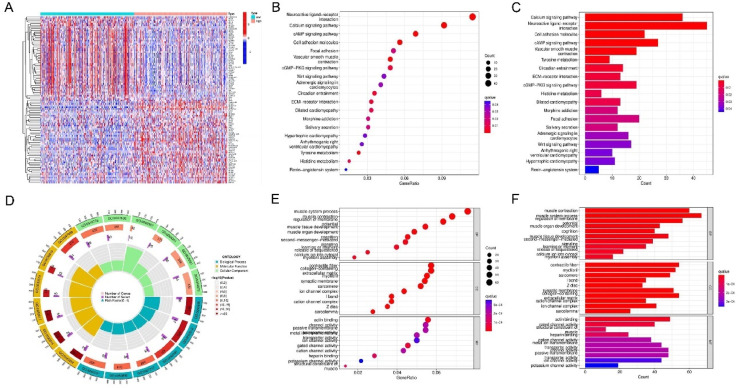
Functional analysis of TRIB3 in LSCC (A) Heatmap of DEGs between high and low TRIB3 expression groups. (B‒C) TRIB3-related KEGG enrichment analysis. (D‒F) TRIB3-related GO function annotation. DEG, Differentially Expressed Genes; GO, Gene Ontology; KEGG, Kyoto Encyclopedia of Genes and Genomes.

LSCC samples were split into two categories according to the TRIB3 expression (high or low). Subsequently, DEGs between them were detected (fdrFilter = 0.05). A total of 1348 differential genes were identified, and a temperature gradient map of the genes were obtained (Fig. 6A). KEGG evaluation revealed that DEG enrichment mainly involved pathways associated with the calcium signaling pathway, the neuroactive ligand-receptor interaction and the cAMP signaling pathway. KEGG assessment highlighted that the enrichment of DEGs mainly involved pathways associated with calcium channels, ligand-neuroactive receptor signaling pathway interplay, and cAMP signaling pathway (Fig. 6B‒6C). GO analysis showed an accumulation of DEGs mainly involved in cellular processes like the muscle system process, cellular components (such as the extracellular matrix myofibril), and molecular functions (such as heparin-binding and antin binding) (Fig. 6D–6F).

### Role of TRIB3 in TME and immune prognosis of LSCC

**Fig. 7:**
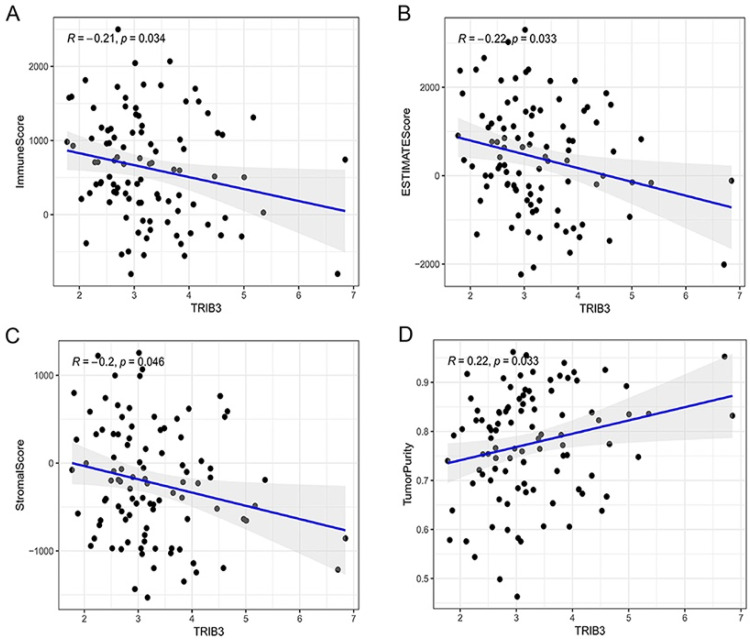
Correlation between tumor microenvironment and TRIB3. (A) Immune Score was negatively correlated with TRIB3 expression; (B) ESTIMATES Score was negatively correlated with TRIB3 expression; (C) Stromal Score was negatively correlated with TRIB3 expression; (D) Tumor Purity was positively correlated with TRIB3 expression (r, Correlation; * p-value < 0.05).

TME refers to the microenvironment composed of tumor vascular, connective tissues with tumor infiltration, and proteins secreted by tumor cells and immune cells. The TIMER survey was conducted to explore common factors affecting relationships between TRIB3 and TME based on the TCGA database. Findings displayed the TRIB3 expression was related to the immune Scoring, ESTIMATES Scoring, stromal Scoring, and the purity of the tumor. Immune Scoring, ESTIMATES score and Stromal score were adversely correlated with TRIB3 expression (*r* < 0, * p-value < 0.05) (Fig. 7A‒7C); Tumor Purity was adversely correlated with TRIB3 expression (*r* > 0, * p-value < 0.05) (Fig. 7D).

**Fig. 8:**
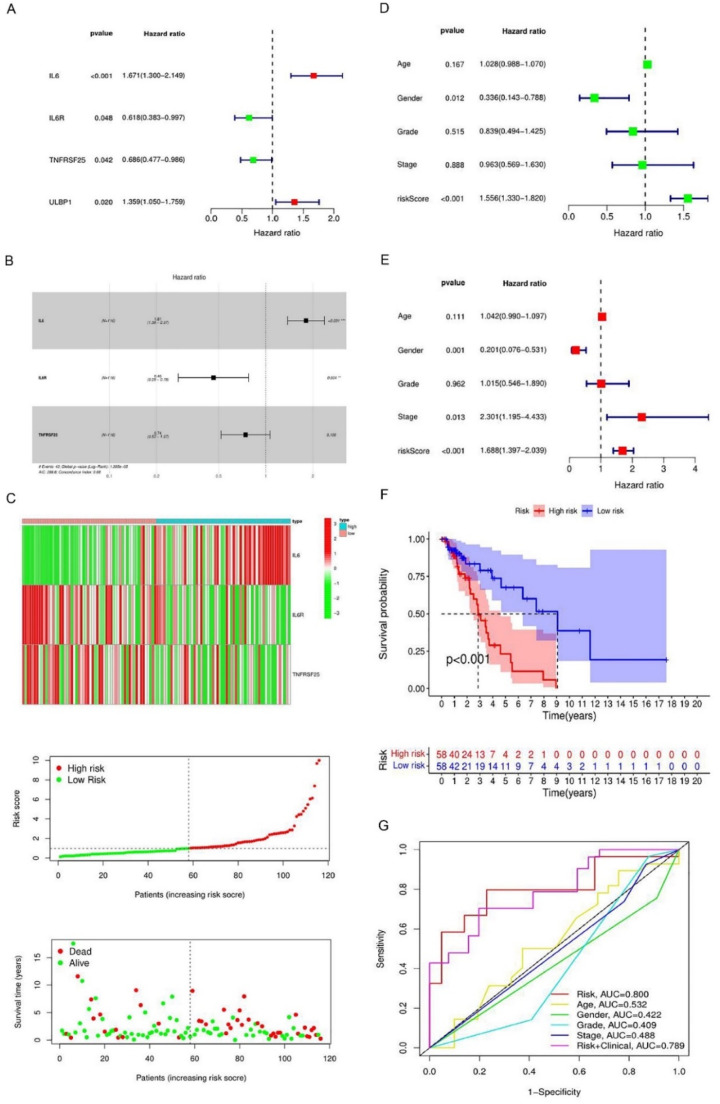
Establishment of immune checkpoint risk model related to TRIB3 in LSCC based on TCGA database. (A) Establishment of prognostic immune checkpoint model related to TRIB3 in LSCC; (B‒C) Establishment of COX risk proportional regression model for TRIB3 and related immune checkpoints (IL6, IL6R, TNFRSF25, ULBP1) in LSCC; (D‒E) Model risk score combined with clinical factors in univariate and multivariate COX analysis; (F) Prognostic analysis showed that the high-risk group had significantly worse prognosis as compared to the low-risk group; (G) ROC curve was employed to evaluate the prediction accuracy of Cox risk model (* p-value < 0.05; ** p-value < 0.01; *** p-value < 0.001).

To further elaborate on the function of TRIB3 for immune prognosis by LSCC, difference expression COX analysis was performed based on the TCGA database, and results showed IL6, IL6R, TNFRSF25, and ULBP1 were the risk factors of prognosis of LSCC (Fig. 8A). In addition, an immunological risk model linked to TRIB3 based on predictive immune modulators was established. The risk score of the model was as follows: riskcore=(coefficient×IL6expansion)+(coefficient×IL6Rexpansion)+(coefficient×TNFRSF25expansion (Fig. 8B). An immunological risk mode was established and samples were classified based on risk and prognosis (Fig. 8C). Univariate Cox analysis showed that age, gender, tumor grade, TNM stage, and risk score were risk factors related to prognosis (Fig. 8D). Univariate Cox analysis showed that aged, sex, tumor stage, TNM status as well as hazard score were risk factors related to prognosis (Fig. 8E). The model showed that the maturation rate was clearly lower in the high-risk group than in the low-risk group (Fig. 8F). An assessment of the accuracy of the ROC curves for the hazard model suggested excellent accuracy for the hazard score of the model (AUC = 0.800) and for the model with constitutional factors (AUC = 0.789) (Fig. 8G). These indicate that TRIB3 mediates the relationship between TIME and poor prognosis of LSCC patients.

## Discussion

Throat cancer is a type of head and neck cancer that is a prevalent Malignancy, representing 30 % of all malignancies of the head and neck. LSCC is the principal pathological form of laryngeal cancer.^12^ Traditionally, LSCC is often managed by radical resection, followed by radiotherapy and chemotherapy depending on the TNM stage. With the in-depth study of signaling pathways related to the TME, immunotherapy has been applied to the therapy of some cancers contains leukemia, breast cancer, prostate cancer, liver cancer, and others.^13^ In the past few years, research has been carried out on TME in head and neck neoplasms (e.g., laryngeal cancer), but little is currently known about TME associated with LSCC. The results of this survey found that prognosis in LSCC was positively associated with immunological status, ESTIMATES, stromal score, and tumor purity in TME, with a particularly negative association with the immunological status score. This implies that the therapy of LSCC is closely tied to relevant TME factors and host immune mechanisms.

TRIB3 is a branch of the Tribbles family of pseudokinases,^14^ which includes TRIB1, TRIB2 and TRIB3.^15^ TRIB3 can not only regulate cell proliferation, differentiation, cell cycle and cell death,^16^^,^^17^ but also modulate the cell cycle and physiological activities of cancer cells.^18^ To date, the part in TRIB3 of the LSCC has no reports available. With the current research, these results displayed TRIB3 overexpression in the LSCC on the basis of TCGA analysis, Western blotting, qRT-PCR and cell functional analysis. According to the survival analysis, methylation site analysis and coexpression analysis of related genes, TRIB3 over-expression was connected to the prognosis for people with LSCC negative. Gene coexpression analysis of the TRIB3 gene was employed to explore the pathways related to TRIB3 expression and a prognostic model was constructed. Model analysis also demonstrated that TRIB3 mediated the poor prognosis of people with LSCC. These findings demonstrate that TRIB3 may act as a profiling biomarker for LSCC and can mediate poor prognosis of LSCC, and that downregulation of TRIB3 exposure can inhibit proliferation, invasion, and metastasis of LSCC. Yet, additional research is necessary to illuminate if TRIB3 can be used as a target for immunotherapy of laryngeal cancer.

The Phosphatidylinositol 3-Kinase (PI3Ks) protein family is a group of intracellular phosphatidylinositol kinases,^19^ and PI3K protein consists of a regulatory substituent, p85, and a facilitative substituent, p110. PI3K protein possesses both The activity of the Serine/Threonine (Ser/Thr) agonist and the phosphatidylinositol agonist.^20^ Its metabolism is related to the products of cancer-causing genes as in v. Src and v. Ras, and it is concerned with the regulation of various cellular processes as in growth, divergence, apoptosis, and glucose transportation.^21^ The up-regulation of PI3K exposure is usually linked to the mechanism of development of different cancers. AKT, also referred to as the Protein Kinase B (PKB), is the primary effector identified downstream of PI3K / AKT can be activated by phosphorylation of PDPK1 and mTORC2, which then activates its substrate rapamycin target protein (mTOR) through both direct and indirect pathways, thereby achieving negative regulation of P13K / AKT sensing channel, which inhibits cell growth and facilitates proliferation and apoptosis.^22^ Research has demonstrated that the PI3K / AKT / mTOR route is one of the principal signaling pathways that regulate glucose metabolism in cancer cells and it is involved in metabolism in some malignant tumors such as hepatocellular carcinoma and so on.^23^ TRIB3 can regulate the PI3K / AKT / mTOR pathway to affect cellular energy metabolism, which has been confirmed in liver cancer, breast cancer, and oropharyngeal cancer.^24^ However, no study has reported whether TRIB3 cancer regulates PI3K / AKT / mTOR pathway to affect the malignant phenotype of LSCC. During the research, GSEA pathway enrichment analysis showed TRIB3 could regulate PI3K / AKT / mTOR pathway to Influence occur, development, and prognosis in LSCC. Then, PPI analysis was conducted for TRIB3 based on the STRING database, and results showed TRIB3 was closely related to the PI3K / AKT / mTOR pathway (a pathway related to AKT). Western blotting demonstrated that the downregulation in TRIB3 exposure suppressed the exposure of PI3K, AKT and mTOR. Taken together, the PI3K / AKT / mTOR pathway is an important pathway mediating the regulatory effects of TRIB3 on LSCC growing, invading and migrating.

Overall, TRIB3 over-expression in the LSCC, which was related to the poor prognosis of LSCC. Patients with methylation related to high TRIB3 expression had a poorer prognosis. Knock-down of TRIB3 expression suppresses the growth, invasion and migration of LSCCs via PI3K / AKT / mTOR. TIME analysis, surface checkpoint analysis, and prediction model indicated that TRIB3 related risk model displayed a poor prognosis. The present research preliminarily explored whether TRIB3 may be viewed as a possible cancer biomarker, which provides a reference for the development of precision medical treatment (such as immunotherapy) of LSCC.

However, in the course of this study, although the regulatory role of TRIB3 on the PI3K / AKT / mTOR pathway in LSCC was confirmed by GSEA pathway enrichment analysis, PPI network analysis, and verified by knockdown of TRIB3 gene expression in some in vitro experiments. However, this study did not perform in vitro experiments of TRIB3 gene overexpression and failed to explore the effect of the TRIB3 gene on LSCC development and progression in vivo in animal models. Therefore, the present study has some limitations. For this reason, based on this study, the authors will explore the specific mechanism of action and targets of TRIB3 in LSCC in future in vitro and in vivo experiments in animal models. In addition, further clinical validation is needed to determine whether the TRIB3 gene is a precise target of LSCC in clinical therapy.

## Abbreviations

TRIB3, Tribbles Pseudokinase 3; TCGA, The Cancer Genome Atlas; LSCC, Laryngeal Squamous Cell Carcinoma; PPI, the Protein-Protein Interaction; CCK8, the Cell Counting Kit-8; PI3K, Phosphatidylinositol 3-Kinases; AKT/PKB, Protein Kinase B; mTOR: the mammalian Target of Rapamycin; TIME, Tumor Immune Microenvironment; OS, Overall Survival time

## Conflicts of interest

The authors declare no conflicts of interest.
